# 外科治疗Ⅲa期小细胞肺癌的疗效分析

**DOI:** 10.3779/j.issn.1009-3419.2017.02.03

**Published:** 2017-02-20

**Authors:** 学军 窦, 志远 王, 亮 王, 伟强 路, 云雷 马, 绍发 许

**Affiliations:** 1 100049 北京，航天中心医院 Department of Thoracic Surgery, Aerospace Central Hospital, Beijing 100049, China; 2 101149 北京，北京市胸科医院胸心外科 Department of Thoracic Surgery, Beijing Chest Hospital, Beijing 101149, China

**Keywords:** 肺肿瘤, 手术治疗, 综合治疗, 预后, Lung neoplasms, Surgical treatment, Comprehensive treatment, Prognosis

## Abstract

**背景与目的:**

小细胞肺癌（small cell lung cancer, SCLC）占所有肺癌的比例近15%左右。SCLC作为一种高度侵袭性肿瘤，恶性程度高，转移早而广泛，对化疗、放疗敏感，初治缓解率高，但容易复发，如果未经任何治疗，其中位生存期仅为4个月-6个月。近年来对SCLC开展了许多研究，但仍未改变SCLC的临床治疗策略，治疗上仍局限于诸如足叶乙甙+顺铂（EP）或卡铂（CE）化疗方案等一些经典的治疗手段，对手术治疗在SCLC治疗，尤其是Ⅲa期的治疗仍未达成共识。本研究旨在探讨Ⅲa期SCLC的外科手术为主的综合治疗对SCLC的临床治疗效果及对影响预后的因素。

**方法:**

通过回顾性分析1995年1月-2010年12月首都医科大学附属北京胸科医院收治手术治疗的78例SCLC患者的临床资料。对患者进行随访，全组病例对性别、年龄、肿瘤大小、淋巴结转移状况、肿瘤-淋巴结-转移（tumor-node-metastasis, TNM）分期、手术方式及术后辅助放化疗进行统计学分析其手术治疗与预后的关系。

**结果:**

本组78例患者中位生存期为13.93个月，术前新辅助化疗47例，中位生存期为14.25个月；术后辅助化疗31例，中位生存期为13.83个月；两者无统计学差异。单站单个（微转移）淋巴结转移28例中位生存期为17.1个月，多站多个淋巴结转移（广泛转移）50例，中位生存期为11.9个月。两者有明显统计学差异（*P* < 0.01）。

**结论:**

进一步评价外科治疗在SCLC综合治疗中的地位及价值，对于Ⅲa期SCLC，以手术为主的综合治疗可以使部分患者受益。

## Abstract (en)

**Background and objective:**

Small cell lung cancer (SCLC) accounts for nearly 15% of all cases of cancer. As a type of highly invasive tumors, SCLC has high degree of malignancy, early and extensive metastasis, and is sensitive to chemotherapy and radiotherapy. The early treatment response rate of SCLC is high but it can also relapse rapidly without any treatment. Its median survival time is merely four to six months. Although many studies on SCLC have been conducted in recent years, its clinical treatment strategies have remained unchanged. The treatment of SCLC is still confined to chemotherapy regimens of etoposide plus cisplatin (EP) and other classic treatments because the surgical treatment of SCLC, particularly for Ⅲa treatment, has yet to reach a consensus. This study investigated the prognostic factors and clinical therapy effect in the comprehensive treatment of Ⅲa SCLC after surgical treatment.

**Methods:**

This study was conducted through the retrospective analysis of the clinical data of 78 patients with SCLC who underwent surgical treatment in Beijing Chest Hospital affiliated to Capital Medical University between January 1995 and December 1995. Through follow-up, we performed statistical analysis of each patient's gender, age, tumor size, lymph node metastasis, tumor-node-metastasis (TNM) staging, surgical methods, and adjuvant radiation and chemotherapy.

**Results:**

The median survival in this clinical trial team was 13.93 months. Among the participants, 47 patients accepted neoadjuvant chemotherapy and their median survival were 14.25 months. By contrast, 31 patients accepted postoperative adjuvant chemotherapy and their median survival were 13.83 months. No statistical difference was observed between the two groups. Moreover, 28 patients were of single Lymph node metastasis and their median survival was 17.1 months. By contrast, 50 patients were of multiple lymph node metastasis and their median survival was 11.9 months. Significant statistical difference was observed between the two groups (*P* < 0.01).

**Conclusion:**

In performing further evaluation of the status and value of surgical treatment in the comprehensive treatment of SCLC, several patients benefitted from Ⅲa SCLC surgery with comprehensive treatment.

肺癌是目前世界最常见的恶性肿瘤之一，其发病率和死亡率逐年上升，在发达国家居癌症死因的首位^[1, 2]^，肺癌已成为我国恶性肿瘤中致死的首要疾病。小细胞肺癌（small cell lung cancer, SCLC）是肺癌的一种特殊类型，占肺癌总数的15%-20%，起源于支气管粘膜上皮和粘液腺内的嗜银细胞，主要特征为肿瘤分化程度低、恶性程度高、倍增时间短等^[3, 4]^。因此，SCLC进展快、容易较早发生淋巴结及全身转移，初始治疗对化疗、放疗较敏感，缓解率较高，但治疗后容易发生耐药，且很容易复发，一旦复发或转移尚没有特别有效的治疗方法^[5, 6]^。20世纪60、70年代以前曾经以手术治疗为主^[7-9]^，在有关外科治疗SCLC的早期报道中，英国医学研究协会开展的外科治疗和放疗的前瞻性随机分组对照研究是最重要的报道之一，其得出的结论是根治性放疗优于化疗^[10-12]^，结果手术被放弃，从那时起放疗成为SCLC的常规治疗，后来许多药物试验用于治疗SCLC，并表现出比较满意的缓解率^[13-15]^，使得后来化疗或化疗+放疗联合模式成为治疗SCLC的常规模式。然而大部分患者在治疗后短期内复发，Lichter等报道化疗+放疗治疗SCLC后，仍有28%-47%病例原发部位复发^[16-18]^，如何有效控制肿瘤局部复发成为了一个重要的问题，使得学者们逐渐又认识到外科手术在治疗期SCLC中所发挥的重要作用。目前国内外专家及学者们对于外科手术在Ⅰ期-Ⅱ期的SCLC治疗中已基本达成共识，但是对于外科手术在Ⅲ期SCLC治疗中的利弊，目前仍存在较大差异^[19-22]^。本文通过回顾我院胸外科治疗SCLC的经验及结合近年来文献对Ⅲa期SCLC外科治疗地位的重新评价进行系统阐述，以便进一步了解SCLC外科治疗的临床研究进展。

## 1 材料与方法

### 1.1 实验对象

通过回顾性分析1995年1月-2010年12月首都医科大学附属北京胸科医院收治手术治疗的78例Ⅲa期SCLC患者的临床资料。所有病例均经术后病理证实为SCLC。病理分期[依据2007年国际抗癌联盟（Union for International Cancer Control, UICC）肿瘤-淋巴结-转移（tumor-node-metastasis, TNM）分期标准]。其中男性65例，女性13例，年龄27岁-75岁，平均51.8岁。

### 1.2 检查

入院后常规行心功能、肝功能、肺功能等检查评估手术风险。留取痰液查癌细胞及支气管镜检查。行胸部增强计算机断层扫描（computed tomography, CT）、头颅磁共振成像（magnetic resonance imaging, MRI）、腹部超声、颈部淋巴结超声及全身骨扫描等检查，排除其他部位转移。

### 1.3 手术治疗

#### 1.3.1 手术时机选择

如术前明确为SCLC患者行化疗2周期后复查胸部CT，如显示肿块影及纵隔淋巴结明显缩小，肺复张、呼吸道症状显著改善。白细胞、血小板、肝肾功能正常后即可行手术治疗。

#### 1.3.2 手术方式

原则上是行肿瘤的根治手术，完整切除肿瘤、最大限度保护健康肺组织。手术方式包括全肺切除48例，肺叶切除30例，其中支气管或（和）肺动脉袖式切除重建8例，所有患者均行系统淋巴结清扫术。

### 1.4 术前、术后辅助治疗方式

#### 1.4.1 新辅助化疗

术前明确为SCLC行术前化疗+术后化疗+放疗。

#### 1.4.2 化疗方案

VP-16+顺铂（或卡铂）为主，化疗4个-6周期。

#### 1.4.3 放疗照射靶区

肺门残端及纵隔，双侧锁骨上淋巴结，脑部放疗等。

### 1.5 随访

所有病例资料均采用信件、电话随访5年。随访率91%。

### 1.6 统计学方法

采用SPSS 19.0进行统计学分析。处理生存率及预后单因素分析采用*Kaplan*-*Meier*法，生存率差异采用*Log*-*rank*法检验，*Cox*回归行多因素预后分析。*P* < 0.05为差异有统计学意义。

## 2 结果

### 2.1 患者预后生存

本组患者中，围手术期死亡2例（术后1个月内死于肺感染和左心衰竭），患者的随访时间为60个月，至随访截止78例患者全部死亡，中位生存期为13.93个月，l年、3年、5年生存率分别为66.7%、29.4%、2.5%（图 1A）。

**1:**
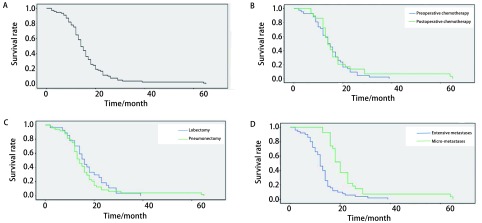
生存曲线。A：患者预后生存曲线；B：术前化疗与术后化疗对比生存曲线；C：肺叶切除与全肺切除对比生存曲线；D：淋巴结微转移与广泛性转移对比生存曲线。 Survival curves. A: Survival curve of patients; B: Comparison of survival curves between preoperative chemotherapy and postoperative chemotherapy; C: Comparison of survival curves between lobectomy and pneumonectomy; D: Comparison of survival curves between micrometastases and extensive metastases of lymph node.

### 2.2 术前化疗与术后化疗生存对比

辅助化疗+放疗：术前化疗+手术+化疗+（或）放疗47例，中位生存期为14.25个月；手术+化疗+（或）放疗31例，中位生存期为13.83个月；两者生存期无明显统计学差异（图 1B）。

### 2.3 肺叶切除与全肺切除生存对比

全肺切除48例，中位生存期为13.3个月；肺叶切除30例，中位生存期为15.2个月。两者生存期无明显统计学差异（图 1C）。

### 2.4 淋巴结微转移与广泛性转移生存对比

单站单个（微转移）淋巴结转移28例中位生存期为17.1个月，l年、3年、5年生存率分别为87.6%、39.3%、10.7%；多站多个淋巴结转移（广泛转移）50例，中位生存期为11.9个月，l年、3年、5年生存率分别为54%、11.2%、0；在生存时间上微转移组要明显长于广泛转移组（*P* < 0.01）（图 1D）。

## 3 讨论

肺癌是当今最常见的恶性肿瘤之一，目前对于肺癌的治疗方法还是传统的手术、化疗和放疗，但是5年生存率仍较低，其中大部分患者一经确诊已是中晚期，失去了最佳治疗机会^[22-24]^。特别是SCLC，由于其病变的特殊性，预后更差。外科治疗经历了争议、矛盾和反复的过程^[25-28]^。对于SCLC的手术治疗特别对于IIb期-Ⅲa期SCLC的患者目前存在很大争议。随着近几年来多学科的合作及综合治疗模式的逐渐成熟，外科参与治疗的SCLC患者生存率得到了明显提高，国内张湘茹、廖美林等^[8, 9]^报道化疗辅助手术治疗局限期SCLC的5年生存率为36%和36.8%，王云杰等^[10]^报道采用术前新辅助化疗，后手术，术后再化疗加或不加放疗的综合治疗方法，5年生存率高达46.5%。但对于Ⅲa期SCLC外科的干预是否使SCLC受益，目前国内外文献报道较少，本组实验表明部分Ⅲa期SCLC患者外科手术干预而受益。

手术时机的选择与术前化疗周期相关，与预后生存时间无关。在Ⅱ期和Ⅲ期的患者中，化疗联合放疗是标准的治疗方式，但也有研究^[29-31]^表明，手术联合辅助化疗同样可以提高生存率及改善预后。有人主张术前化疗至少3个-4个周期。我们认为过多的化疗会引起骨髓严重的抑制，白细胞和血小板的降低，造成凝血机制破坏，肝肾功能不全等并发症。而且纠正这些并发症需要花费较长时间，致使化疗中断，手术延迟甚至失去手术机会。另外胸部影像学上阴影的消失并不代表肺组织内无癌细胞^[32-34]^。本组资料显示新辅助化疗与生存时间无明显关系。

手术方式的选择与预后生存时间无关。我们选择手术方式的原则是尽可能地进行根治性切除，周围型病变行肺叶切除+纵膈淋巴结清扫，中心型病变则根据病变范围，选择全肺切除者或行袖式切除以保证术后良好的肺功能。本组资料显示术式与术后并发症、切缘癌残留以及肺功能相关，生存时间无明显关系。

单站单个（微转移）淋巴结转移生存时间明显长于多站多个淋巴结转移（广泛转移）。本组单因素和多因素分析显示N2转移组数是影响预后的重要因素，单组N2转移的1年、3年、5年生存率分别为87.6%、39.3%、10.7%。两组以上N2转移的1、3、5年生存率分别为54%、11.2%、0（*P* < 0.01）。N2淋巴结转移个数与生存率有关（*P* < 0.01），本组资料显示对于淋巴结广泛转移的SCLC患者预后较差，手术干预对于这部分患者受益较小。

与非小细胞肺癌（non-small cell lung cancer, NSCLC）相比，长期以来人们大多认为外科手术治疗SCLC效果较差，而放化疗效果较好。但国内外许多研究表明手术治疗结合化疗+放疗对于SCLC仍能达到比较好的效果。但是IIb期-Ⅲa期患者是否适合手术仍存在较大的争议。由于新辅助化疗的毒副作用，术前应该进行化疗的次数疗程，是否应根据不同的化疗药物制定不同周期的术前化疗疗程，这方面的研究很少^[35]^。由于对SCLC来说，化疗是主要的治疗方法。手术治疗主要目的是切除原发灶，防止复发，减轻瘤负荷，那么楔形手术是否更有利于患者，而不是目前依照NSCLC的手术方式行肺叶切除+纵隔淋巴结清扫，目前涉及此方面的研究较少^[36]^。总之，外科手术作为SCLC多学科综合治疗的一部分，其作用越来越明显，根据患者的机体状况、肿瘤的临床分期以及对各种治疗的耐受程度具体选择，如何采取以外科手术为主的综合治疗，患者从各种治疗中最大程度的获益，才是我们需要进一步研究的问题^[37-39]^。
